# Dangers of Middle Class Restaurants, Coffee Stalls and Some Public Houses

**Published:** 1919-05-31

**Authors:** 


### "THE POISON IN THE CUP."

### Dangers of Middle Class Restaurants, Coffee Stalls and some Public Houses.

Whilst eating a hasty meal in a very busy middle-class restaurant near one of our large London railway stations, we chanced to witness certain cleansing rites being performed upon the establishment's eating utensils. The technique of this operation was of quite spartan simplicity. Knives, forks, and spoons were put into a species of large jam pot and the plates into a tub. No steam rose from either receptacle. When necessary a knife or fork was taken out of the pot, hastily wiped on a cloth, and duly presented to a customer.

To see is to believe, and we must say that this little experience surprised us. Thirsting for further knowledge, we sought a coffee-stall at midnight, an hour of busy custom, and was again amazed to find that the only cleansing process observed was a hasty dip of the soiled article into an enamelled bowl of water, followed by a hasty rub with a damp towel, which was common to all the utensils wiped. Now a very eager crowd clamoured round this stall, the attendants working at high pressure to supply their needs. The supply of crockery was scanty and frequent washings required, everything being performed at break-neck speed.

On another occasion we visited a public-house in a densely populated area near London Bridge at the busy time of the day, the bar being packed to the doors with a thirsty motley crowd all eager to be served. In this instance the glasses were used over and over again, the cleansing process being a mere dip into a bowl of cold water and a hasty wipe with a cloth.

We protest vigorously against such proceedings, for if the authorities give licences or rights to publicans and suchlike to serve the public, they should also protect the public weal. For reasons of common decency and cleanliness alone, we would insist upon the proper washing of eating and drinking utensils, but in addition there is allowed to exist a grave risk in spread of communicable disease.

A proportion of our population suffer from syphilis, and a proportion of these have the disease in its communicable stage. Such people are free to use our public eating and drinking places and to spread infection if defective hygiene is allowed to flourish in the manner we have indicated. To give a case in point: after a visit to a venereal clinic we visited a restaurant nearby, and there also refreshing herself was a young woman whom we had seen at the clinic half an hour previously. She was suffering from severe secondary syphilis, and had been given a dated card of admission to hospital for doses of intravenous salvarsan.

This question of hygiene we refer to our Ministry of Health or other constituted authority, and suggest that a proper supervision be exercised over the cleanliness of our public eating and drinking places. Knives, forks, and the like should be thoroughly washed in boiling water, preferably containing a dilute inocuous antiseptic, and afterwards wiped on a clean cloth. The imposition of inspection and supervision would be quite an easy matter, and of definite public service, both in preventing spread of contagious disease, and in impressing the moral effect of cleanliness upon the poorer classes of our population.

